# Dose-dependent metabolite changes after ethanol intoxication in rat prefrontal cortex using *in vivo* magnetic resonance spectroscopy

**DOI:** 10.1038/s41598-019-47187-4

**Published:** 2019-07-23

**Authors:** Louise Carton, Florent Auger, Maeva Kyheng, Maud Pétrault, Nicolas Durieux, Delphine Allorge, Olivier Cottencin, Renaud Jardri, Régis Bordet, Benjamin Rolland

**Affiliations:** 1 University Lille, Inserm U1171 ‘Degenerative and vascular cognitive disorders’, F-59000 Lille, France; 2 CHU Lille, department of Pharmacology, F-59000 Lille, France; 3 CHU Lille, Psychiatry and Addiction Medicine Department, F-59000 Lille, France; 4 University Lille, Preclinical Imaging Core Facility, F-59000 Lille, France; 5 Univ. Lille, EA 2694 - Santé publique : épidémiologie et qualité des soins, F-59000 Lille, France; 6 CHU Lille, Service de Biostatistiques, F-59000 Lille, France; 7 CHU Lille, Unité Fonctionnelle de Toxicologie, F-59000 Lille, France; 8 University Lille, EA 4483 - IMPECS - IMPact de l’Environnement Chimique sur la Santé humaine, F-59000 Lille, France; 9 University Lille, CNRS UMR 9193 SCALab PsyCHIC Team, F-59000 Lille, France; 10 CHU Lille, Psychiatry Department, CURE platform, Fontan Hospital, F-59000 Lille, France; 11 Service Universitaire d’Addictologie de Lyon (SUAL), Pôle MOPHA, CRNL, Inserm U1028, CNRS UMR5292, Université Lyon 1, Centre Hospitalier Le Vinatier, Bron, France

**Keywords:** Brain, Addiction, Preclinical research

## Abstract

Ethanol disrupts the balance between the excitatory (glutamatergic) and inhibitory (GABAergic) neurotransmission systems. We aimed to assess how acute ethanol intoxication in rats affects the levels of GABA, glutamate and other cerebral metabolites after injection of two different doses of ethanol. One *in vivo* magnetic resonance spectrum of the prefrontal cortex region was acquired before and six spectra were acquired after intraperitoneal injections of saline or ethanol (1 g/kg or 2 g/kg). Brain kinetics after exposure to ethanol were compared to blood ethanol kinetics. GABA levels significantly decreased after injection of 1 g/kg but not 2 g/kg doses of ethanol. Choline levels, which serve as a marker of alterations in membrane composition, significantly decreased after injection of 2 g/kg but not 1 g/kg doses of ethanol. Acute ethanol intoxication appears to result in specific dose-dependent changes in the GABA level and choline level.

## Introduction

The high clinical variability in the cognitive and behavioral disruptions induced by ethanol may be due to its distinctive patterns of action on inhibitory (GABAergic) and excitatory (glutamatergic) neurotransmission. GABA is the main inhibitory neurotransmitter of the central nervous system in mammals^1^. In conjunction with glutamate, it regulates the inhibition/excitation balance that causes long-term depression and long-term potentiation modulations, which modify synaptic transmissions. This balance has an important role in the brain plasticity necessary for regulation of behavioral, cognitive, memory and learning functions^1^. A previous study found that acute ethanol intoxication activates GABA interneurons and may act on glutamatergic projection neurons of the prefrontal cortex^2^. A better understanding of the relationship between ethanol concentration and its effects on the GABAergic and glutamatergic systems in the prefrontal cortex appears to be crucial to account for and manage the complex effects of acute ethanol intoxication. Indeed, the prefrontal cortex is particularly involved in the alteration of cognitive function related to acute ethanol intoxication^3^.

Magnetic resonance spectroscopy (MRS) is a noninvasive technique for studying biological systems *in vivo*. MRS allows assessment of the levels of different metabolites, including *N-*acetylaspartate (NAA), creatine, choline and glutamate, in the brain. NAA is a marker related to neuronal activity and integrity^4^. Changes in choline levels are generally associated with alterations in membrane composition^4^. Creatine is considered a stable metabolite and is commonly used as a reference concentration^4^. Interestingly, ethanol has also been detected on MR spectra in both humans and animals^5–11^, while recent technical developments now allow the identification of GABA levels^12,13^. MRS can therefore detect *in vivo* changes in GABAergic and glutamatergic neurotransmission and in neuronal integrity with NAA and choline metabolites.

A limited number of previous MRS studies have assessed the impact of acute ethanol administration on the *in vivo* levels of cerebral metabolites in rats^5,7,8,13,14^. Furthermore, none have included the GABA signal in metabolites assessed within the prefrontal cortex.

In this context, the purpose of the present study was to carry out kinetic modeling of the modulation of GABA and glutamate levels in response to acute ethanol intoxication in rats induced by two different doses (1 g/kg and 2 g/kg).

## Results

### Ethanol kinetic aspects

**Figure 1:**
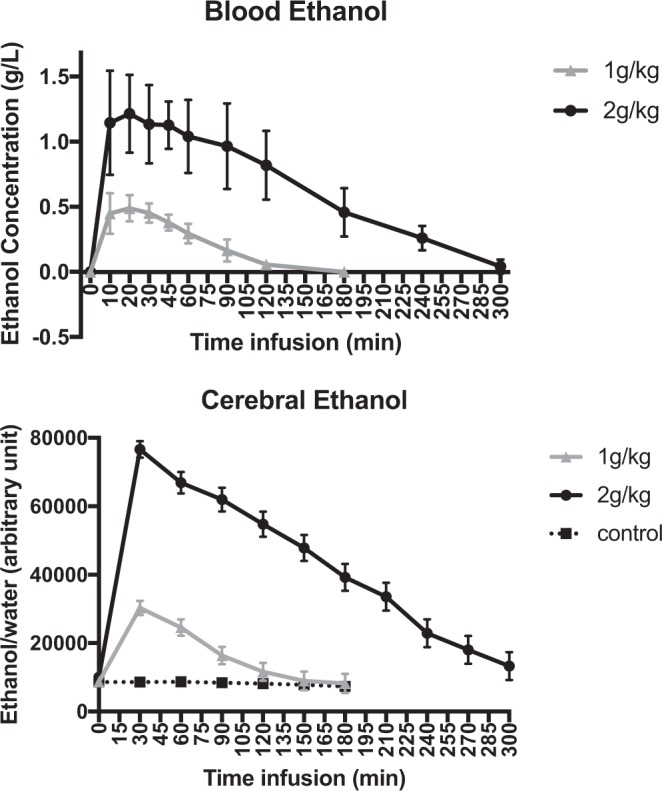
Kinetics of blood and cerebral ethanol after ip administration of 1 g/kg and 2 g/kg doses of ethanol. The middle values represent the mean blood concentration of ethanol in g/L (above) and the mean level of cerebral ethanol in arbitrary units (below). The error bar represents the standard deviation (SD).

We obtained full kinetic data for 3 animals in each group Fig. 1.

In both groups, the peaks of ethanol in the blood and within the prefrontal cortex occurred within the first 30 minutes after intraperitoneal (ip) ethanol administration (Fig. 1). At peak time, the blood alcohol concentration was 1.21 ± 0.3 g/L in the 2 g/kg group and 0.49 ± 0.1 g/L in the 1 g/kg group and then dropped below 0.1 g/L after 120 minutes in the 1 g/kg group and after 300 minutes in the 2 g/kg group.

### Prefrontal metabolite levels

#### GABA

**Figure 2:**
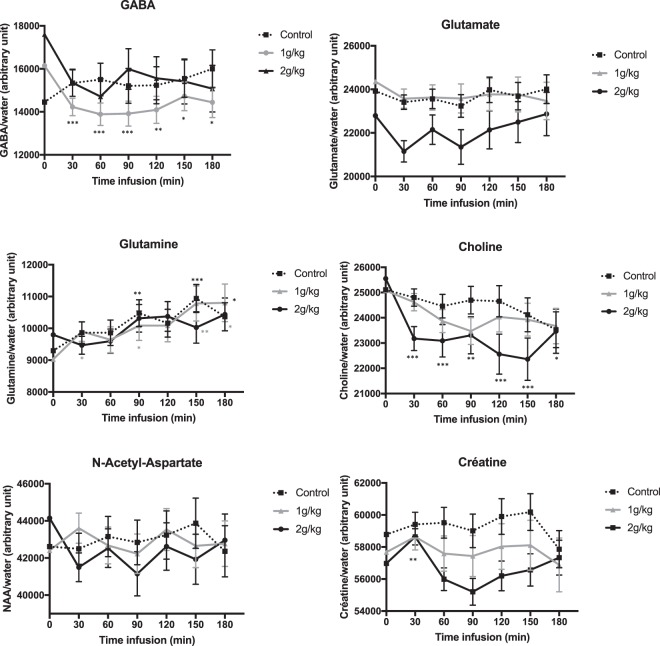
Change from initial basal level of the metabolites after either ethanol (1 g/kg and 2 g/kg groups) or saline administration. The middle values represent the mean level of metabolite after each ethanol treatment, and the error bar represents the standard deviation. The variations were significantly different from the baseline values (*0.05 < p-value < 0.009; **0.009 < p-value < 0.0009; ***0.0009 < p-value < 0).

**Figure 3:**
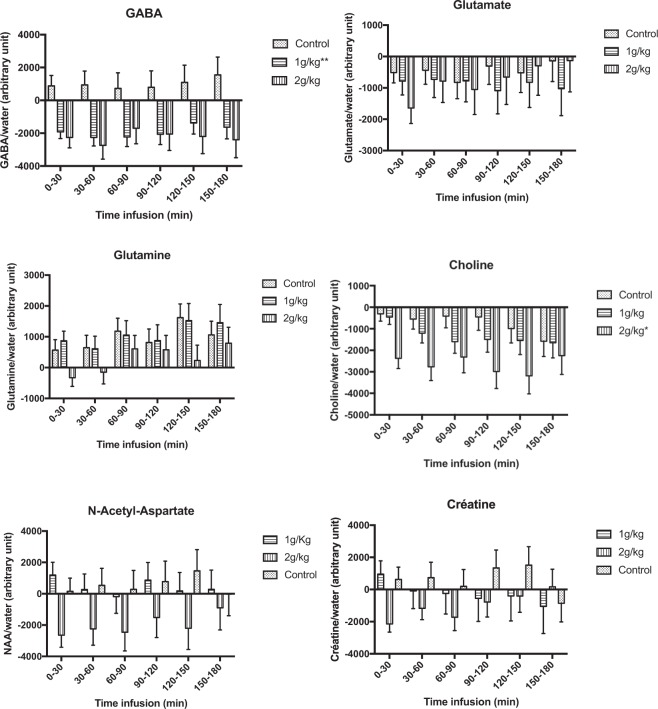
Comparisons of the change from initial basal level of metabolites between the 1 g/kg and control groups, between the 2 g/kg and control groups, and between the 1 g/kg and 2 g/kg groups. The middle values represent the mean level of variation in metabolite after each ethanol intoxication, in comparison to baseline level and the error bar represents the standard deviation. The differences were significant in comparison to the control group (*0.05 < p-value < 0.009; **0.009 < p-value < 0.0009; ***0.0009 < p-value < 0).

GABA levels decreased significantly with increased time in the 1 g/kg ethanol group (global time effect, p < 0.001) but not in the 2 g/kg ethanol (p = 0.070) or control (p = 0.67) groups. Compared to baseline, GABA levels in the 1 g/kg group decreased by 12% within the first 30 minutes, and this decrease was observed until 180 minutes after ethanol administration (p < 0.05 for all *post hoc* pairwise comparisons, Fig. 2). The variation in GABA levels of this group was significantly different from that measured in the control group, without a significant ‘time*group’ interaction (p = 0.94) Figs 2 and 3.

#### Glutamate

No changes in the levels of glutamate were observed over time.

#### Glutamine

Glutamine levels increased significantly with increasing time in the 1 g/kg ethanol group (global time effect p = 0.026) and control group (p = 0.022) but not in the 2 g/kg ethanol group (p = 0.084). However, the comparison of the change from the initial basal level between both groups did not show a significant difference.

#### Choline

Choline levels significantly decreased with increasing time in the 2 g/kg ethanol group (global time effect p = 0.0002) but not in the 1 g/kg ethanol (p = 0.14) or control (p = 0.31) groups. Compared to baseline, choline levels in the 2 g/kg group decreased by 9% within the first 30 minutes, and this decrease was observed until 180 minutes after ethanol administration (p < 0.05 for all *post hoc* pairwise comparisons, Fig. 2). The variation in choline levels observed in this group was significantly different from that measured in the control group, without a significant ‘time*group’ interaction (p = 0.23).

#### Creatine

Creatine levels significantly decreased with increasing time in the 2 g/kg ethanol group (global time effect p = 0.003) but not in the 1 g/kg ethanol group (p = 0.75) or the control group (p = 0.16). Compared to baseline, creatine levels in the 2 g/kg group decreased by 4% within the first 30 minutes, but this decrease did not persist to 180 minutes after ethanol administration (p > 0.05 for all *post hoc* pairwise comparisons, Fig. 2), except at 90 minutes (p = 0.047). However, this change was not significantly different from that observed in the control group.

#### NAA

No changes in the levels of NAA over time were observed.

## Discussion

To our knowledge, our study is the first to model the *in vivo* kinetic effects of acute ethanol intoxication on the inhibitory/excitatory balance in rats, with particular reference to the GABA spectrum in the prefrontal cortex. We used two different doses of ethanol, namely, 1 g/kg and 2 g/kg, and observed state-dependent changes in the levels of different brain metabolites (GABA and choline) as a function of ethanol intoxication.

For both ethanol doses, the kinetics in the blood and brain were relatively similar, with an ethanol concentration peak occurring within the first 30 minutes. By comparison, a 0.49 g/L and 1.21 g/L blood alcohol concentration of ethanol in humans corresponds approximately to an intake of 2 or 3 standard units of alcohol and to an intoxication by binge-drinking in an adult, respectively. We found dose-specific effects of ethanol on GABA levels, i.e., a significant reduction at a dose of 1 g/kg but no significant effect at a dose of 2 g/kg. Previous preclinical data have remained controversial. For instance, reduced GABAergic neurotransmission was previously found *via* metabolic models of the cortex in murine brains after ethanol (2.5 g/kg) intoxication^11^. Conversely, another *ex vivo* study showed increased GABA levels in the rat prefrontal cortex after injection of 1.5 g/kg ethanol compared to 2.5 g/kg ethanol^15^. Our finding of a reduction in brain GABA levels induced by ethanol in the 1 g/kg group was, nevertheless, consistent with the results of previous *in vivo* studies that focused on the human occipital cortex after mild, acute ethanol intoxication^10^ or on the prefrontal cortex of adults after alcohol intoxication due to binge-drinking^16^. Such reductions in GABA levels were previously proposed to be linked to the potentiation of GABA receptors by ethanol, thus triggering a reduced need for GABA transmission^10^. If correct, this hypothesis suggests that changes in GABA levels would be mediated by a reduction in GABA-synthetizing enzyme, i.e., glutamic acid decarboxylase or by a decrease in GABA release from vesicles. Crucially, suppression of glutamic acid decarboxylase activity was previously observed in the rodent hypothalamus after ethanol intoxication^17^. Macroscopically, a decrease in GABA levels would be consistent with low-dose behavioral effects. Since the prefrontal cortex is essential for executive function^18^, GABAergic potentiation could account for the behavioral disinhibition observed in the first stage of acute ethanol intoxication. The lack of effect on GABA levels following 2 mg/kg intoxication may be linked to a dose-dependent effect on the GABAergic system. At low doses, ethanol intoxication could have an impact on the most sensitive GABA interneurons whereas the higher dose effect may correspond to complex GABA-glutamate interactions in the prefrontal cortex.

The significant decrease in choline observed in the 2 g/kg group could reflect membrane adaptation that occurs in response to high doses of ethanol. This decrease was concordant with the reduced levels of choline observed in murine^5^ and human brains after acute ethanol intoxication^10^, although both previously observed decreases occurred after lower ethanol intoxication and did not reach significance after Bonferroni correction. However, contradictory results have shown either an increase in choline levels after ethanol intoxication in murine models^7,8,14^ or no variation^15^. Our study did not find any significant change in the NAA levels over time but observed a decreasing trend only in the 2 g/kg group. Taken together, the observed decrease in choline and the trend towards a decrease in NAA levels at higher doses of ethanol could be markers of the dose-dependent cerebral intoxication present in a drunk person.

Significant changes in the levels of creatine were observed in the 2 g/kg group. Interestingly, in MRS studies, creatine has often been considered a stable metabolite and has been used as a reference concentration^4^. However, some previous studies found a decrease in the levels of creatine, whereas other studies did not find any significant change after ethanol administration^5,9,10^. Altogether, these findings support our choice of using water rather than creatine as an internal reference for the ratio calculations.

Two main methodological issues need to be acknowledged. The first is inherent to the current MRS procedures since we had no means of disentangling the intracellular from extracellular metabolites in the data acquired. Therefore, the decreased GABA levels might have resulted from (i) a decreased number of GABAergic neurons, (ii) smaller neurons, or (iii) decreased extracellular GABA levels^19^. However, the first two hypotheses seem unlikely to occur after a short time exposure. The most likely explanation appears to be a decrease in extracellular GABA levels following an influence of release. These interpretations are further limited by the predefined region of interest that was used for the MRS acquisition. The second limitation concerns isoflurane use as a potential confounding factor. However, by using a control group, we ensured that no significant variations in the metabolite levels could be attributed to isoflurane.

## Conclusion

Using an *in vivo* exploratory method, we found dose-dependent modulation of GABA levels in the prefrontal cortex after acute ethanol intoxication. Impairments in neuronal integrity were also observed at higher doses. This information should be used in conjunction with other noninvasive clinical imaging methods, neuropathological studies, and behavioral animal studies to achieve a more complete understanding of the pathogenesis related to ethanol.

## Materials and Methods

### Blood kinetics procedure (experiment 1)

#### Animals

Thirteen wild-type male Wistar rats (Janvier Laboratories) (aged 10–15 weeks; mean weight, 357 g) were used for the experiments according to a protocol approved by the French ethics committee *Comité d’Ethique en Experimentation Animale Nord-Pas de Calais n°75*. Before the experiments, the rats were maintained for ten days under conditions of constant temperature and humidity and a 12 hour light/dark cycle, with free access to food.

#### Schedule

After the stabilization period, the rats were anesthetized with isoflurane (3% induction) to allow the placement of an intrajugular catheter. Subsequently, the rats were assigned to different ethanol groups and received intraperitoneal (ip) injections of either 1 g/kg (N = 4) or 2 g/kg (N = 9) doses of ethanol. Blood samples were collected at 10, 20, 30, 45, 60, 90, 120 and 180 minutes after injection for the 1 g/kg group and at 10, 20, 30, 45, 60, 90, 120, 180, 240 and 300 minutes after injection for the 2 g/kg group. The amount of blood drawn from the rats at each time interval was 200μL for both low and high doses. The blood ethanol concentration was determined using an ISO 15189-2012 validated headspace gas chromatography with a flame ionization detector (HS-GC-FID) method that was adapted from previously published protocols^20,21^. The linear range of the assay was 0.05–5.0 g/L.

### MRS procedure (experiment 2)

#### Animals

Experiments 1 and 2 used independent samples. Thirty wild-type male Wistar rats (Janvier Laboratories) (aged 10–15 weeks; mean weight, 380 g) were used for the experiments according to a protocol approved by the CEEA 75 *(Comité d’Ethique en Experimentation Animale)*. Before the experiments, the rats were maintained for ten days under conditions of constant temperature and humidity and a 12 hour light/dark cycle, with free access to food.

#### Schedule

After the stabilization period, rats were anesthetized with isoflurane (3% induction) to allow the placement of an ip catheter. Baseline MRS scan acquisition was performed before the ip injection. Each rat was then assigned to one of three groups that received different doses of ethanol: (a) a 1 g/kg dose of ethanol (N = 10) group, (b) a 2 g/kg dose of ethanol (N = 10) group, and (c) a saline control (N = 10) group. Several MRS acquisitions were then repeated until 3 hours after the treatments.

#### Anesthesia and monitoring

Animals had a nose cone for the delivery of the isoflurane anesthesia (1.5–3%) and oxygen (1.5 L/min). The blood-oxygen saturation, pulse rate, rectal temperature and respiration of each rat were monitored throughout the experiment.

#### MRI acquisition

**Figure 4:**
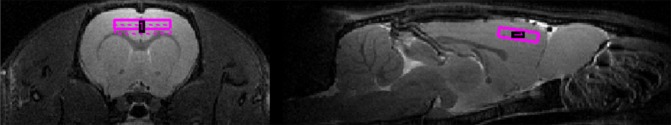
Voxel localization in the prefrontal cortex.

Experiments were performed on a 7.0 Tesla Animal Biospec MR Scanner (Bruker, Ettlingen, Germany). Two coils were used: a cylindrical transmitter coil with a 72 mm internal diameter and a surface coil, which was placed on the scalp of each animal for signal reception. Gradient echo acquisition was first carried out to confirm the placement of the animal in the apparatus. T2-weighted anatomical sequences were then carried out in the axial and sagittal planes to position the MRS acquisition voxel (1 * 6 * 4 mm^3^) in the prefrontal cortex^22^ (Fig. 4**)**. The acquisition parameters were as follows: TR/TE = 2500/33 ms, squared field of view = 4 cm, encoded by a squared matrix of 256 and 16 slices of 0.5 mm.

#### MRS acquisition and postprocessing

**Figure 5:**
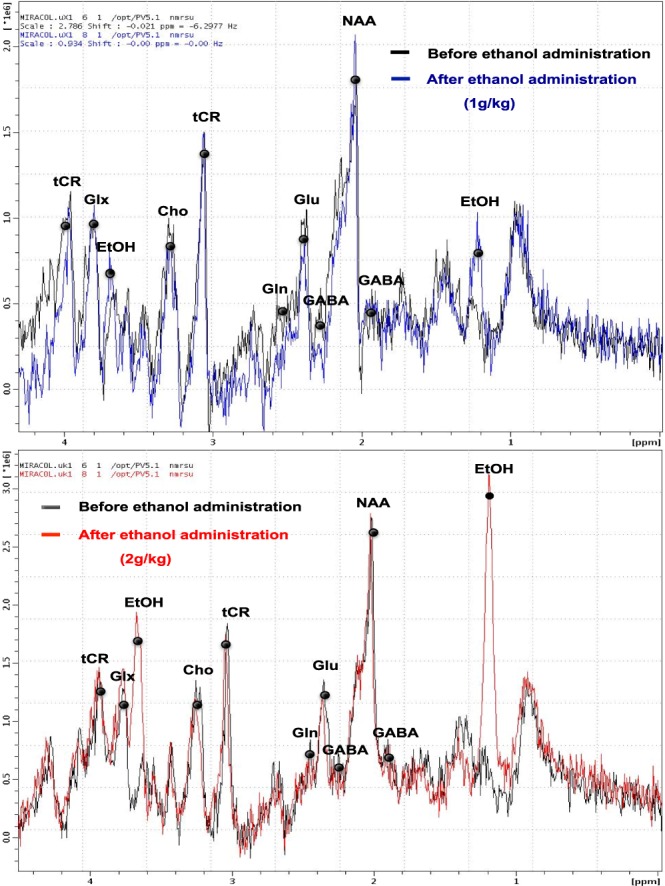
Spectra obtained before (black line) and after ip administration of ethanol at 1 g/kg (blue line) and 2 g/kg (red line).

Unsuppressed and suppressed STimulated Echo Acquisition Mode (STEAM; TR/TE = 3500/3 ms, NEX = 512) sequences were performed. Before each MR unsuppressed acquisition a second-order shim was performed. Water suppression was accomplished using a VAriable Pulse power and Optimized Relaxation delays (VAPOR) technique^23^ and no outer volume suppression module was used. Water peaks obtained with unsuppressed water acquisitions were used (1) to check the spectral resolution and (2) as an internal reference to estimate metabolite quantity. The water signal of the tissue was chosen as the adjustment parameter because the levels of creatine showed substantial longitudinal variations among the different groups. The integrated area under the curve was used for quantification. The Signal Noise Ratio (SNR) of spectra for the control group, 1 g/kg group and for the 2 g/kg group were respectively the following: 28.48 ± 4.14; 27.59 ± 5.20 and 28.02 ± 0.93. The linewidth values of water peak were 11.53 ± 1.10 Hz in the control group; 11.48 ± 1.04 Hz in the 1 g/kg group and 13.01 ± 1.48 Hz in the 2 g/kg group. The quality of the spectrum allowed evaluation of the signal for the following metabolites: GABA (1.9 and 2.28 ppm), glutamate (glu) (2.35 ppm), glutamine (gln) (2.45 ppm), ethanol (EtOH) (1.18 and 3.66 ppm), NAA (2.02 ppm), choline (cho) (3.22 ppm) and total creatine (cre) (3.03 and 3.92 ppm) (Fig. 5).

Postprocessing of the MRS data was performed using JMRUI 5.1 software^24^, while the AMARES algorithm^25^ was used for quantification of the main metabolites.

### Statistical analysis

We assessed the changes in the levels of different metabolites within the three groups separately over time compared to baseline using linear mixed models (LMMs). LMMs allow handling the correlations between the repeated measurements within the same subject by using a covariance pattern model with a first-order autoregressive covariance structure (since the measurements were taken at predetermined and evenly spaced intervals). In these LMMs, only time was considered as a fixed effect; in the case of significant time effects, post hoc pairwise comparisons with baseline values were performed using linear contrasts. We also used LMMs with a first-order autoregressive covariance structure to compare metabolite variations from the baseline of each ethanol group with the control group. These models included time, group, time*group interaction and baseline value as fixed effects. In the case of significant time*group interactions, post hoc pairwise comparisons between groups at each time point were performed using linear contrasts; otherwise, the interaction term was removed from the model to estimate the average group effect. Data were analyzed using SAS software version 9.4 (SAS Institute, Cary, NC).

## References

[1] Govindpani, K. *et al*. Towards a Better Understanding of GABAergic Remodeling in Alzheimer’s Disease. *Int. J. Mol. Sci*. **18**, (2017). 10.3390/ijms18081813 PMC5578199 28825683

[2] Leriche M, Méndez M, Zimmer L, Bérod A (2008). Acute ethanol induces Fos in GABAergic and non-GABAergic forebrain neurons: a double-labeling study in the medial prefrontal cortex and extended amygdala. Neuroscience.

[3] Heinz AJ, Beck A, Meyer-Lindenberg A, Sterzer P, Heinz A (2011). Cognitive and neurobiological mechanisms of alcohol-related aggression. Nat. Rev. Neurosci..

[4] Govindaraju V, Young K, Maudsley AA (2000). Proton NMR chemical shifts and coupling constants for brain metabolites. NMR Biomed..

[5] Adalsteinsson E, Sullivan EV, Mayer D, Pfefferbaum A (2006). *In vivo* quantification of ethanol kinetics in rat brain. Neuropsychopharmacol. Off. Publ. Am. Coll. Neuropsychopharmacol..

[6] Biller A, Bartsch AJ, Homola G, Solymosi L, Bendszus M (2009). The effect of ethanol on human brain metabolites longitudinally characterized by proton MR spectroscopy. J. Cereb. Blood Flow Metab. Off. J. Int. Soc. Cereb. Blood Flow Metab..

[7] Zahr NM (2010). Brain injury and recovery following binge ethanol: evidence from *in vivo* magnetic resonance spectroscopy. Biol. Psychiatry.

[8] Zahr NM (2013). A mechanism of rapidly reversible cerebral ventricular enlargement independent of tissue atrophy. Neuropsychopharmacol. Off. Publ. Am. Coll. Neuropsychopharmacol..

[9] Liu H (2014). Acute ethanol-induced changes in edema and metabolite concentrations in rat brain. BioMed Res. Int..

[10] Gomez R (2012). Intravenous ethanol infusion decreases human cortical γ-aminobutyric acid and N-acetylaspartate as measured with proton magnetic resonance spectroscopy at 4 tesla. Biol. Psychiatry.

[11] Tiwari V, Veeraiah P, Subramaniam V, Patel AB (2014). Differential effects of ethanol on regional glutamatergic and GABAergic neurotransmitter pathways in mouse brain. J. Neurochem..

[12] Puts NAJ, Edden RAE (2012). *In vivo* magnetic resonance spectroscopy of GABA: a methodological review. Prog. Nucl. Magn. Reson. Spectrosc..

[13] Mullins PG (2014). Current practice in the use of MEGA-PRESS spectroscopy for the detection of GABA. NeuroImage.

[14] Zahr NM (2016). Transient CNS responses to repeated binge ethanol treatment. Addict. Biol..

[15] Lee D-W (2014). Dose-dependent influence of short-term intermittent ethanol intoxication on cerebral neurochemical changes in rats detected by *ex vivo* proton nuclear magnetic resonance spectroscopy. Neuroscience.

[16] Silveri MM (2014). Altered anterior cingulate neurochemistry in emerging adult binge drinkers with a history of alcohol-induced blackouts. Alcohol. Clin. Exp. Res..

[17] Seilicovich A (1985). The effect of acute ethanol administration on GABA receptor binding in cerebellum and hypothalamus. Eur. J. Pharmacol..

[18] Funahashi S, Andreau JM (2013). Prefrontal cortex and neural mechanisms of executive function. J. Physiol. Paris.

[19] Levy LM, Degnan AJ (2013). GABA-based evaluation of neurologic conditions: MR spectroscopy. AJNR Am. J. Neuroradiol..

[20] Corrêa CL, Pedroso RC (1997). Headspace gas chromatography with capillary column for urine alcohol determination. J. Chromatogr. B. Biomed. Sci. App..

[21] Cheung ST, Lin WN (1987). Simultaneous determination of methanol, ethanol, acetone, isopropanol and ethylene glycol in plasma by gas chromatography. J. Chromatogr..

[22] Paxinos, G. & Watson, C. The rat brain in stereotaxic coordinates, 7th edition. In (Academic Press, 2013).

[23] Tkác I, Starcuk Z, Choi IY, Gruetter R (1999). *In vivo* 1H NMR spectroscopy of rat brain at 1 ms echo time. Magn. Reson. Med..

[24] Naressi A (2001). Java-based graphical user interface for the MRUI quantitation package. Magma N. Y. N.

[25] Vanhamme L, van den Boogaart A, Van Huffel S (1997). Improved method for accurate and efficient quantification of MRS data with use of prior knowledge. J. Magn. Reson. San Diego Calif 1997.

